# Different Interactive Effects of Metformin and Acarbose With Dietary Macronutrient Intakes on Patients With Type 2 Diabetes Mellitus: Novel Findings From the MARCH Randomized Trial in China

**DOI:** 10.3389/fnut.2022.861750

**Published:** 2022-04-26

**Authors:** Yu An, Yinhui Li, Nannan Bian, Xiaoyu Ding, Xiaona Chang, Jia Liu, Guang Wang

**Affiliations:** Department of Endocrinology, Beijing Chao-Yang Hospital, Capital Medical University, Beijing, China

**Keywords:** type 2 diabetes mellitus, macronutrients, drug-diet interaction, MARCH trial, acarbose and metformin

## Abstract

Antidiabetic oral agents and nutrition management are frequently used together as first-line therapies for type 2 diabetes mellitus (T2DM). However, less is known about their interaction. The interactive effect of two classic antidiabetic medications, namely, acarbose and metformin, with dietary intakes of macronutrients on glycemic control and cardiometabolic risk factors was investigated in the metformin and acarbose in Chinese as the initial hypoglycemic treatment (MARCH) randomized clinical trial. The patients with newly diagnosed T2DM from China were included in the trial. Participants were randomized to receive either metformin or acarbose monotherapy as the initial treatment, followed by a 24-week treatment phase, during which add-on therapy was used if necessary. Dietary intakes of carbohydrate, protein, fat, and total energy were calculated by a 24-h food diary recall method. Linear mixed-effect models combined with a subgroup analysis were used to investigate independent and interactive effects of drugs and diet on clinical outcomes. A data analysis was performed on 551 of the 788 patients randomly assigned to treatment groups. Metformin therapy was independently associated with higher triglycerides (TGs, β = 0.471, *P* = 0.003), 2 h postprandial plasma glucose (2hPPG, β = 0.381, *P* = 0.046) but lower low-density lipoprotein cholesterol (LDL-C, β = −0.149, *P* = 0.013) compared with acarbose therapy. Higher carbohydrates and lower fat intakes were independently associated with poorer glycemic control, less weight loss, and greater insulin secretion. Higher total energy intake was also independently associated with higher fasting (β = 0.0002, *P* = 0.001) and postprandial blood glucose (β = 0.0004, *P* = 0.001). Interaction and subgroup analyses demonstrated that glucagon-like peptide-1 (GLP-1) was positively related to total energy (β = 0.268, *P* = 0.033), carbohydrates intake, and insulin secretion (β = 2,045.2, *P* = 0.003) only in the acarbose group, while systolic blood pressure (SBP) was negatively related to protein intake in the metformin group (β = 23.21, *P* = 0.014). The results of this study showed that metformin and acarbose mainly exerted different interactive effects with dietary energy, carbohydrate, and protein intakes on GLP-1 secretion, insulin release, and SBP. The interaction between drug therapy and nutrition intervention in glycemia highlights the complexity of combination therapy.

## Introduction

The number of patients with type 2 diabetes mellitus (T2DM) is increasing annually, which poses a global health threat and causes high health care costs, especially in the Asian region (1). It has been reported that the prevalence of diabetes in Asian countries, ranging from 3 to 47%, continues to surge despite therapeutic advances, with more than 60% of patients with diabetes worldwide living in Asia (2). T2DM is also reaching epidemic proportions in China, a country with the largest number of patients with diabetes (3), and thus there is an urgent need for more effective or targeted treatment strategies for it.

Nowadays, the treatment for T2DM is based on lifestyle changes, especially diet and antidiabetic oral agents, according to the needs of the patients. The treatment aims to achieve and maintain the optimal glycemic control, prevent acute diabetic complications, and reduce the risk of chronic complications, which require a combination of diabetic diet and conventional medications (4). Although numerous clinical trials are helpful in comparing different treatment strategies or medication regimens, the results of these trials should be considered in combination with patients' specific conditions, the availability and cost of drugs, and physicians' judgment in decision-making.

Metformin has been recommended as a first-line drug for monotherapy or combination therapy by Western authoritative clinical practice guidelines when lifestyle interventions can no longer achieve glycemic control (5). In China, acarbose has been taken as one of the first-line drugs in treatment for diabetes because acarbose can slow down the digestion and absorption of dietary carbohydrates in small intestines by inhibiting brush-border α-glucosidase, and Chinese diet is characterized by a higher percentage of carbohydrates (6). Zhu et al. have confirmed that the hypoglycemic effect of acarbose in patients with T2DM consuming an Eastern diet is superior to that in those consuming a Western diet by a systematic meta-analysis of studies (7). Because of the specific mechanism of glycemic control and weight loss, dietary components may alter the hypoglycemic effect of acarbose.

In fact, in addition to pharmacological interventions, dietary interventions are another cornerstone therapy for diabetes prevention and management (8, 9). The essential therapeutic approach to reducing the incidence and severity of T2DM focuses on the nature and quality of nutrients, especially consumed energy and macronutrients (10). The macronutrients in the diet are utilized by the body as sources of energy, including carbohydrate, protein, and fat. Restriction on any one of these macronutrients will have to be compensated by increase in the proportion of energy derived from the other two (11). Diets have evolved with changes in time, cultural traditions, as well as geographic and economic factors, all of which interact in a complex manner to shape different dietary consumption patterns among countries and regions (12). It has been reported that most Asians follow an Eastern diet pattern, characterized by higher intakes of whole grains, legumes, vegetables, fruits, and fish, thus making dietary carbohydrates the major source of energy (13). Evidence has suggested that the macronutrient composition of the diet also plays a key role in the postprandial and diurnal glucose excursions in patients with T2DM, and its changes may contribute to reducing postprandial glycemia, HbA1c, and diabetic complications (14).

However, there is a lack of information on Chinese dietary patterns and the efficacy of acarbose. The metformin and acarbose in Chinese as the initial hypoglycemic treatment (MARCH) trial was thus conducted to compare the effects of acarbose and metformin as the initial therapy for Chinese patients newly diagnosed with T2DM. It became the first and unique trial to investigate the effect of dietary macronutrients on glycemic control in the context of intensive drug therapy (ClinicalTrial.gov, number ChiCTR-TRC-08000231) (15–17). The trial showed that acarbose has a similar efficacy to metformin, making it a viable option for Chinese patients. Nevertheless, no previous studies of drug–diet interaction were conducted by systematic analysis of the diet and clinical data from MARCH. In this study, data from the MARCH trial were used to examine whether the effects of two classic antidiabetic medications, namely, acarbose and metformin, on glycemic control and cardiometabolic risk factors are related to dietary macronutrient intakes in patients with T2DM.

## Materials and Methods

### Study Design

The study design, preliminary results, and the full study protocol of the MARCH trial were previously published (15). The MARCH trial was a multicenter (11 clinical sites in China), randomized controlled clinical trial in patients newly diagnosed T2DM. In the trial, the changes in the dietary intakes of energy and macronutrients, HbA1c, and fasting plasma glucose (FPG), postprandial 2-h glycemic profile, body mass index (BMI), insulin, glucagon, GLP-1, and insulin sensitivity or β-cell function by homoeostatic model assessment (HOMA) index in patients were evaluated. These patients received sustained-released metformin hydrochloride up to 1,500 mg, one time per day (500 mg per tablet, Beijing Double Crane Pharma, Beijing, China), or up to 100 mg of acarbose, three times per day (50 mg per tablet, Bayer Healthcare, <city>Beijing</city>, China), and there will be a 24-week monotherapy and a 24-week insulin secretagogue add-on therapy if necessary. At Chinese diabetic clinics, outpatients who met the inclusion criteria were provided with written notification and oral explanations on this trial. This study began after the acquisition of written informed consent and confirmation of willingness to participate from all the participating patients. The protocol was approved by the ethics committee from each clinical site and implemented in accordance with the provisions of the Declaration of Helsinki and Good Clinical Practice guidelines.

### Participants

**Figure 1:**
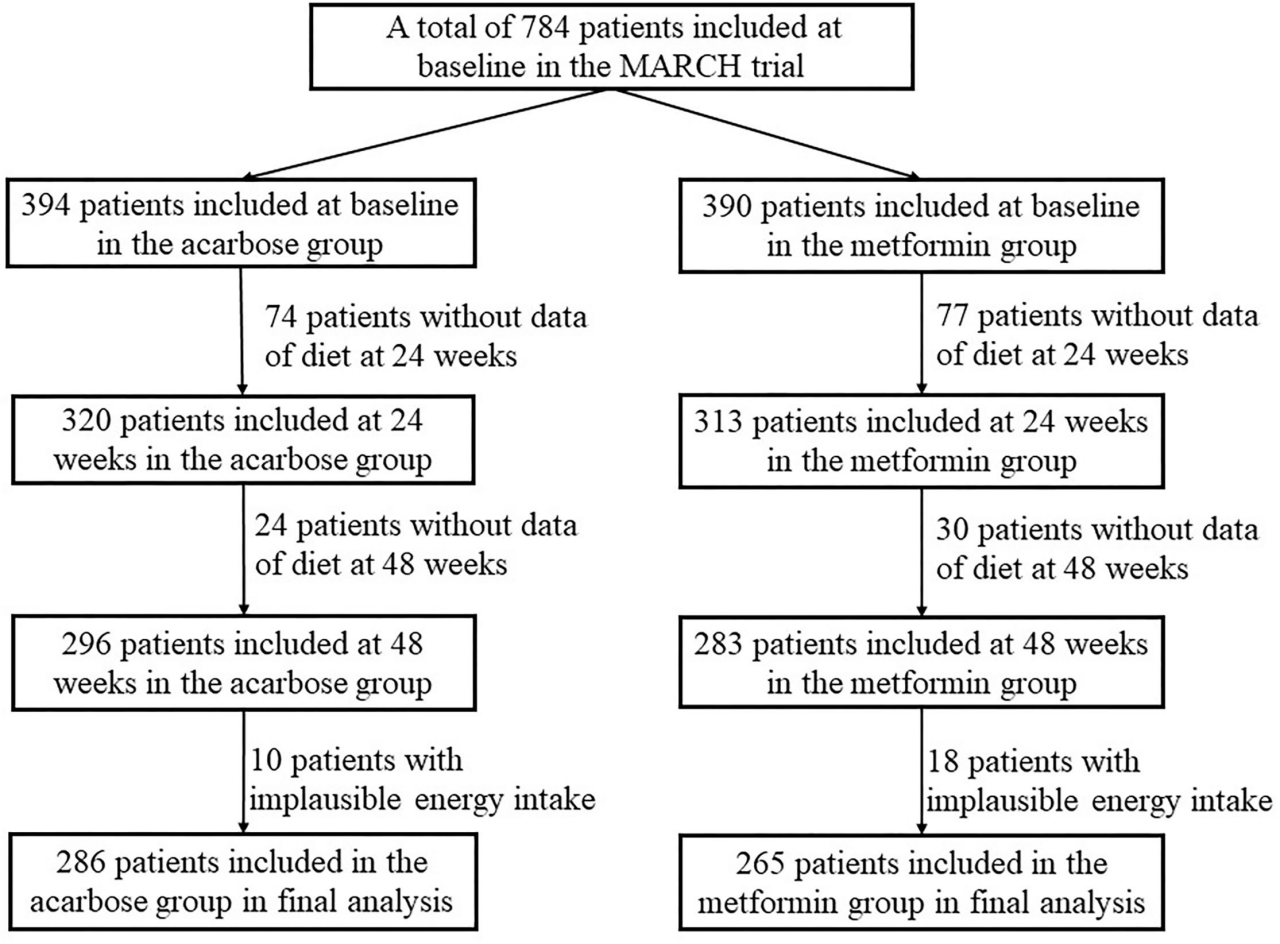
Flowchart for the selection process of the current study population in the metformin and acarbose in Chinese as the initial hypoglycemic treatment (MARCH) trial.

Patients would be eligible for this study if they fulfilled the following criteria: (1) diagnosed with T2DM within the past 12 months according to 1999 WHO criteria, (2) without oral antidiabetic agents or short-term (i.e., 1 month) treatment discontinued 3 months before enrollment, and (3) suboptimum glucose control (HbA1c between 7.0% and 10.0%, FPG ≤ 11·1 mmol/L, and BMI 19–30 kg/m^2^). The full details of exclusion criteria were previously described. During the recruitment phase, 788 eligible individuals were invited to participate. Four participants withdrew consent before drug intervention and were excluded. Subsequently, 98 participants were excluded from the acarbose group and 107 from the metformin group due to insufficient dietary data. In addition, 10 participants in the acarbose group and 18 in the metformin group with implausible energy intake (<800 kcal/day or >6,000 kcal/day for men; <600 kcal/day or >4,000 kcal/day for women) were further excluded (18). Finally, a total of 551 participants (286 in the acarbose group and 265 in the metformin group) were included in this study. A flowchart of the selection process of the study population is shown in Figure 1.

### Dietary Assessment

Dietary macronutrient intake and energy intake were assessed at baseline, 24 and 48 weeks by the 24-h dietary recall method, and an author's semiquantitative diet history questionnaire, in which questions concern the quantity and the frequency of product consumption in each group. The questionnaire has been reviewed by clinical nutritionists and dietitians (19). A well-trained dietitian conducted face-to-face interviews with the patients and asked them to report an average portion size of commonly consumed food. Food models, pictures, and other visual presentations were used to designate portion sizes of food and thus guide participants in estimating the portion size of consumed food. Additionally, a pilot study was performed on several patients and modified accordingly. The food data in the questionnaires were matched with nutrient information from China Food Composition Database for the analysis of macronutrient content. All data of food intake frequencies were converted to times per day, and then the portion size of each food item per day as well as the daily energy and macronutrient intakes from it were calculated and derived. The percentage of total kilocalories from carbohydrate, protein, and fat intakes was then derived.

### Outcomes and End Points

The primary end points were reduction in HbA1c at 24 and 48 weeks. Key secondary end points included changes in fasting blood glucose (FBG), 2-h postprandial glycemic profile, BMI, insulin, glucagon, glucagon-like peptide-1 (GLP-1), and insulin sensitivity or β-cell function (HOMA index), which were all measured at baseline, 24, and 48 weeks. The glucose metabolism variables included HbA1c, FBG, 2-h postprandial plasma glucose (2hPPG), HOMA of insulin resistance (HOMA-IR), and whole-body insulin sensitivity index (WBISI). Hormone secretion parameters included fasting insulin (FINS), HOMA of β-cell function (HOMA-β), early insulin secretion index (EISI, I_30_/G_30_), and the area under the curve (AUC) of insulin, glucagon, and GLP-1. The measurement of cardiometabolic risk factors related to obesity, dyslipidemia, and high blood pressure included height, weight, serum levels of total cholesterol (TC), low-density lipoprotein cholesterol (LDL-C), high-density lipoprotein cholesterol (HDL-C) and triglycerides (TGs), systolic blood pressure (SBP), and diastolic blood pressure (DBP). BMI was calculated from the measured height and weight (kg/m^2^). The following formulas were used to calculate some of the indexes mentioned above: HOMA-IR = FINS (μIU/ml) × FBG (mmol/L)/22.5; HOMA-B = 20 × FINS (μIU/ml)/[FBG (mmol/L) −3.5] early insulin secretion index (EISI) = ΔI_30_/ΔG_30_ = (insulin_30min_ – insulin_0min_)/ΔG_30_ (glucose_30min_ – glucose_0min_); and WBISI = 10,000/square root of [(mean plasma insulin × mean plasma glucose during OGTT) × (FINS × FPG). The AUC was calculated with the following equations: AUC for serum insulin = (insulin_0min_ + insulin_30min_) × 30/2 + (insulin_30min_ + insulin_120min_) × 90/2 + (insulin_120min_ + insulin_180min_) × 60/2, AUC for glucagon= (glucagon _0min_ + glucagon _30min_) × 30/2 + (glucagon _30min_ + glucagon _120min_) × 90/2 + (glucagon _120min_ + glucagon _180min_) × 60/2 and AUC for plasma GLP-1 = (GLP-1_0min_ + GLP-1_30min_) × 30/2 + (GLP-1_30min_ + GLP-1_120min_) × 90/2 + (GLP-1_120min_ + GLP-1_180min_) × 60/2.

### Statistical Analysis

Statistical analyses were performed by STATA version 13.0 (STATA, College Station, TX) and R 3.5.1. Prior to analysis, the normality of data distribution was checked. Continuous variables were expressed as medians (interquartile range, IQR) when nonnormally distributed, and they were the mean ± standard deviation (SD) when normally distributed. The Student's *t*-test or the Mann-Whitney *U*-test was used for continuous variables as appropriate at baseline. The differences in frequencies of the categorical variables were evaluated using the chi-square test. The Friedman's two-way analysis of variance by ranks test was adopted to determine changes in treatment outcomes and dietary intakes of energy and macronutrients in two treatment groups during three time periods (i.e., baseline, 24 weeks, and 48 weeks). This nonparametric test was used since the data did not meet the requirements for parametric analysis. When the difference was significant (Friedman's test, *P* < 0.05), *post-hoc* pairwise comparison tests with a Bonferroni correction were performed, and scatter plots were used to visually display changes over time. Multilevel mixed-effect linear regression models were adopted (maximum likelihood method, unstructured variance-covariance structure, and center and individual used as random effect components) to determine the independent associations between dietary energy and macronutrient intakes or between two antidiabetic oral drug classes and treatment outcomes over time. Furthermore, to analyze the effect of the drug–diet interaction between acarbose or metformin treatment and dietary energy and macronutrients intakes on treatment outcomes, interaction terms of drug class were added to each intake of nutrient in the adjusted linear mixed-effect regression models, respectively. In addition, subgroup analysis was performed on acarbose or metformin treatment groups to test the significant interactive associations of dietary energy and macronutrient intakes and treatment outcomes in each group. Coefficients (β) were adopted to express the effect size of the associations. All effect size estimates were adjusted for covariates, including age, sex, time of intervention, and duration of diabetes. A two-sided *P* < 0.05 showed statistical significance.

## Results

### Participant Characteristics

Demographic, anthropometric, and clinical characteristics of the patients with diabetes receiving different treatments are shown in Table 1. There were 286 patients in the acarbose group and 265 in the metformin group. The median age of the 551 participants was 51 (IQR: 43–57), of which 218 were women (39.6%). Among them, the patients only in the metformin group had significantly longer duration of diabetes (*P* = 0.039). There were no statistically significant differences in age, sex, glucose metabolism variables, hormone secretion parameters, and cardiometabolic risk factors between participants (all *P* > 0.05).

**Table 1:** Baseline characteristics of patients treated with acarbose or metformin.

**Variables**	**Acarbose**	**Metformin**	***P*** **value**
	**(*****n*** **= 286)**	**(*****n*** **= 265)**	
Sex			0.748
Male, *n*%	171 (59.79%)	162 (61.13%)	
Female, *n*%	115 (40.21%)	103 (38.87%)	
Age, years	51 (44, 57)	51 (43, 57)	0.672
Duration of diabetes	0.13 (0.09, 0.26)	0.16 (0.09, 0.30)	0.039^*^
BMI, kg/m^2^	25.26 (23.39, 27.12)	25.26 (23.65, 27.38)	0.853
SBP, mmHg	121 (118, 130)	120 (120, 130)	0.853
DBP, mmHg	80 (70.5, 82)	80 (73, 82)	0.917
TC, mmol/L	5.12 (4.46, 5.84)	5.10 (4.32, 5.93)	0.755
TG, mmol/L	1.80 (1.23, 2.71)	1.96 (1.38, 2.76)	0.166
HDL-C, mmol/L	1.18 (1.02, 1.39)	1.18 (1.00, 1.37)	0.712
LDL-C, mmol/L	3.07 (2.53, 3.59)	3.02 (2.43, 3.63)	0.359
FBG, mmol/L	8.2 (7.5, 9.1)	8.3 (7.6, 9.5)	0.197
HbA1c, %	7.2 (6.6, 8.1)	7.5 (6.7, 8.4)	0.108
2hPPG^$^, mmol/L	12.44 (10.55, 14.40)	12.58 (10.23, 14.80)	0.796
FINS, uIU/mL	11.06 (7.24, 16.58)	11.71 (7.24, 16.83)	0.733
EISI^$^	2.54 (1.08, 4.48)	2.59 (1.13, 4.49)	0.902
AUC for plasma GLP-1^$^, pmol/mL × min	2,797.35 (1,753.20, 4,427.85)	2,829.68 (1,716.30, 4,884.60)	0.628
AUC for glucagon^$^, pg/mL × min	12,256.35 (9,936.15, 15,887.93)	12,353.10 (9,730.20, 15,813.34)	0.922
AUC for serum insulin^$^, uIU/mL × min	4,528.50 (3,198.00, 6,236.70)	4,435.05 (3,017.48, 6,160.95)	0.437
HOMA-IR	3.87 (2.44, 6.30)	4.09 (2.56, 6.60)	0.606
HOMA-β	48.58 (29.13, 77.62)	51.43 (28.25, 73.06)	0.985
WBISI^$^	3.83 (2.64, 5.85)	3.85 (2.63, 5.88)	0.947

*Data shown as median (interquartile range) were compared between 2 groups using Mann-Whitney U-test*.

*Data shown as n (%) were compared between 2 groups using the chi-square test*.

*BMI, body mass index; SBP, systolic blood pressure; DBP, diastolic blood pressure; TGs, triglycerides; TC, total cholesterol; LDL-C, low-density lipoprotein cholesterol; HDL-C, high-density lipoprotein cholesterol; FBG, fasting blood glucose; 2hPPG, 2-h postprandial plasma glucose; FINS, fasting insulin; HOMA-IR, homeostasis model assessment of insulin resistance; HOMA-β, homeostasis model assessment of β-cell function; EISI, early insulin secretion index; WBISI, whole body insulin sensitivity index; AUC, area under the curve; GLP-1, plasma glucagon-like peptide-1*.

$ *After a standard meal test*.

* *P < 0.05*.

### Changes in Clinical Outcomes and Dietary Intakes Over Time

**Figure 2:**
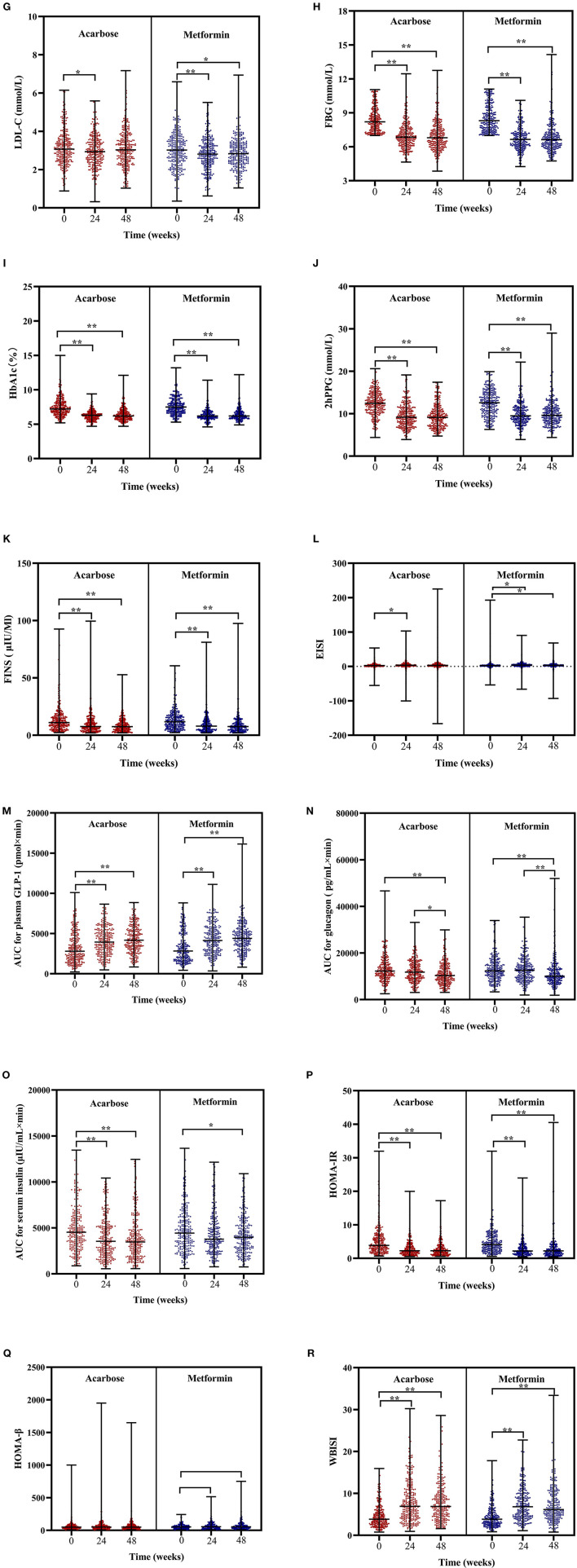
Scatter plots of BMI **(A)**, SBP **(B)**, DBP **(C)**, TC **(D)**, TG **(E)**, HDL-C **(F)**, LDL-C **(G)**, FBG **(H)**, HbA1c **(I)**, 2hPPG **(J)**, FINS **(K)**, EISI **(L)**, AUC for plasma GLP-1 **(M)**, AUC for glucagon **(N)**, AUC for serum insulin **(O)**, HOMA-IR **(P)**, HOMA-β **(Q)**, and WBISI **(R)** in patients subjected to metformin or acarbose groups at baseline, 24 weeks, and 48 weeks. Data were tested by Friedman's two-way analysis of variance by ranks test during three time periods (i.e., baseline, 24 weeks, and 48 weeks) in two treatment groups. *post-hoc pairwise comparison adjusted *P* < 0.05, ** *post-hoc* pairwise comparison adjusted *P* < 0.01. BMI, body mass index; SBP, systolic blood pressure; DBP, diastolic blood pressure; TGs, triglycerides; TC, total cholesterol; LDL-C, low-density lipoprotein cholesterol; HDL-C, high-density lipoprotein cholesterol; FBG, fasting blood glucose; 2hPPG, 2-h postprandial plasma glucose; FINS, fasting insulin; HOMA-IR, homeostasis model assessment of insulin resistance; HOMA-β, homeostasis model assessment of β-cell function; EISI, early insulin secretion index; WBISI, whole body insulin sensitivity index; AUC, area under the curve; GLP-1, plasma glucagon-like peptide-1.

The Friedman's two-way analysis of variance by ranks combined with *post-hoc* pairwise comparisons indicated that there was the significant effect of overall treatment time on most treatment outcomes. The scatter plots of *post-hoc* pairwise comparison revealed that the BMI, TC, FBG, HbA1c, 2hPPG, FINS, AUC for plasma GLP-1, HOMA-IR, and WBISI significantly decreased in both treatment groups at 24 and 48 weeks compared with those at baseline. SBP significantly increased at 48 weeks compared with that at 24 weeks, while DBP significantly decreased at 24 weeks compared with that at baseline only in acarbose group. TG significantly decreased at 24 and 48 weeks compared with that at baseline only in the acarbose group. LDL-C in the acarbose group significantly decreased at 24 weeks compared with that at baseline but significantly decreased both at 24 and 48 weeks compared with that at baseline in the metformin group. Meanwhile, the change trend of EISI in two groups was opposite to that of LDL-C. AUC for glucagon significantly decreased at 48 weeks compared with that at baseline and at 24 weeks in both treatment groups. AUC for serum insulin significantly decreased at 24 and 48 weeks compared with that at baseline in the acarbose group but significantly decreased at 48 weeks compared with that at baseline in the metformin group (adjusted *P* < 0.05, Figures 2A–R).

**Figure 3:**
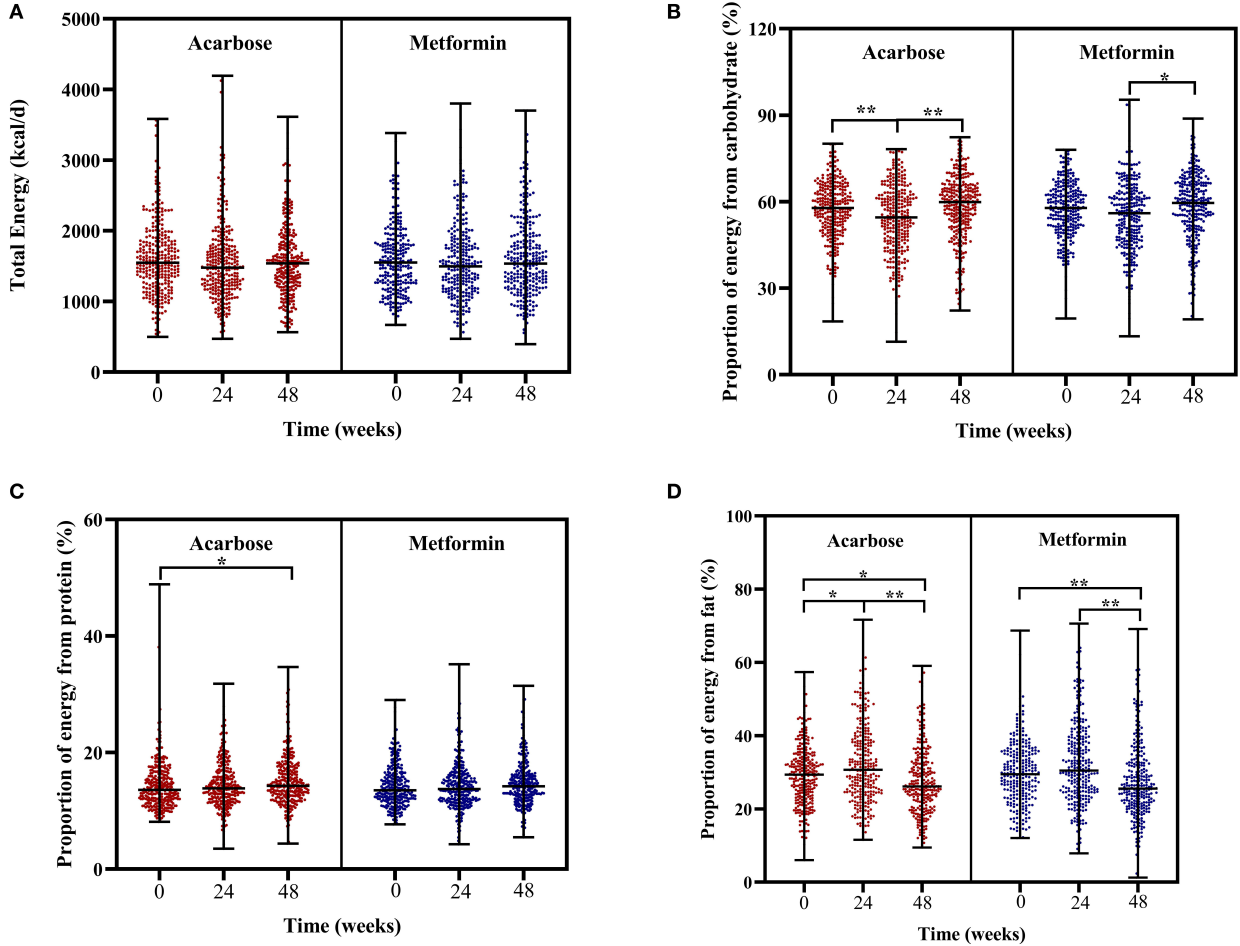
Scatter plots of total energy intake **(A)**, proportion of energy from carbohydrate **(B)**, proportion of energy from protein **(C)**, and proportion of energy from fat **(D)** in patients subjected to metformin or acarbose groups at baseline, 24 weeks, and 48 weeks. Data were tested by Friedman's two-way analysis of variance by ranks test during three time periods (i.e., baseline, 24 weeks, and 48 weeks) in two treatment groups. *post-hoc pairwise comparison adjusted *P* < 0.05, **post-hoc pairwise comparison adjusted *P* < 0.01.

In regard to changes in dietary intakes of total energy and macronutrients, the Friedman's two-way analysis of variance by ranks indicated that there was a significant effect of overall treatment time on proportion of energy from carbohydrate, protein, and fat. *Post-hoc* pairwise comparisons showed the following results: the proportion of energy from carbohydrate significantly decreased at 24 weeks compared with that at baseline but significantly increased at 48 weeks compared with that at 24 weeks in the acarbose group and in the metformin group; the proportion of energy from carbohydrate only significantly increased at 48 weeks compared with that at 24 weeks; the proportion of energy from protein significantly increased at 48 weeks compared with that at baseline only in the acarbose group; the proportion of energy from fat significantly increased at 24 weeks compared with that at baseline only in the acarbose group but significantly decreased at 48 weeks compared with that at 24 weeks and at baseline in both groups (adjusted *P* < 0.05, Figures 3A–D).

### Independent or Interactive Associations of Treatment Effects and Dietary Intakes With Clinical Outcomes

Linear mixed-effect models were used to assess the effects of acarbose or metformin treatment on changes in clinical parameters adjusted for age, sex, time of intervention, and disease duration. Results were expressed as estimated mean changes from baseline with 95% confidence intervals (CIs). In drug treatment effects, metformin therapy was independently associated with higher TG (0.471 mmol/L, 95% CI: 0.159–0.782 mmol/L), higher 2hPPG (0.381 mmol/L, 95% CI: 0.007–0.756 mmol/L), and lower LDL-C (0.149 mmol/L, 95% CI: −0.266 to −0.032 mmol/L) but had comparable effects on other outcomes compared with acarbose therapy (Table 2). Dietary intakes of energy, carbohydrate, and fat also demonstrated independent associations with certain clinical outcomes (Table 3). Each unit increase in total energy intake was significantly associated with higher FBG (0.002 mmol/L, 95% CI: 0.00008–0.0003 mmol/L) and higher 2hPPG (0.004 mmol/L, 95% CI: 0.0002–0.0007 mmol/L). Each unit increase in proportion of energy from carbohydrate was significantly associated with higher BMI (0.607 kg/m^2^, 95% CI: 0.167–1.047 kg/m^2^), higher HbA1c (0.685%, 95% CI: 0.251–1.120%), higher 2hPPG (1.255 mmol/L, 95% CI: 0.103–2.407 mmol/L), and higher AUC for serum insulin (1,090.1 μIU/ml × min, 95% CI: 176.6–2,003.6 μIU/ml × min). In contrast, each unit increase in proportion of energy from fat was significantly associated with lower BMI (0.808 kg/m^2^, 95% CI: −1.314 to −0.303 kg/m^2^), lower FBG (0.770 mmol/L, 95% CI: −1.370 to −0.170 mmol/L), lower HbA1c (0.969%, 95% CI: −1.476% to −0.462%), lower 2hPPG (1.954 mmol/L, 95% CI: −1.476 to −0.462 mmol/L), lower AUC for serum insulin (1,135.1 μIU/ml × min, 95% CI: −2,193.8 to −76.4 μIU/ml × min) but higher WBISI (2.673, 95% CI: 0.059–4.758).

**Table 2:** Independent effects of drug treatment on clinical outcomes in linear mixed-effect models.

**Outcomes**	**Acarbose**	**Metformin**	
		**β (95%CI)**	***P*** **value**
BMI, kg/m^2^	Ref	0.149 (−0.267–0.565)	0.483
SBP, mmHg		−0.049 (−1.490–1.392)	0.947
DBP, mmHg		0.060 (−0.840–0.959)	0.897
TC, mmol/L		−0.021 (−0.169–0.128)	0.786
TG, mmol/L		0.471 (0.159–0.782)	0.003^*^
HDL-C, mmol/L		−0.198 (−0.059–0.020)	0.323
LDL-C, mmol/L		−0.149 (−0.266 −0.032)	0.013^*^
FBG, mmol/L		−0.086 (−0.229–0.056)	0.236
HbA1c, %		0.041 (−0.080–0.163)	0.506
2hPPG, mmol/L		0.381 (0.007–0.756)	0.046^*^
FINS, μIU/Ml		0.035 (−1.007–1.077)	0.947
EISI		0.357 (−1.135–1.849)	0.639
AUC for plasma GLP-1, pmol × min		134.3 (−48.2–316.8)	0.149
AUC for glucagon, pg/mL × min		182.0 (−483.2–847.2)	0.592
AUC for serum insulin, μIU/mL × min		170.1 (−136.8–477.1)	0.277
HOMA-IR		0.033 (−0.330–0.396)	0.857
HOMA-β		−3.021 (−12.492–6.451)	0.532
WBISI		−0.271 (−0.810–0.268)	0.324

*Models adjusted for age, sex, time of intervention, and duration of diabetes*.

*BMI, body mass index; SBP, systolic blood pressure; DBP, diastolic blood pressure; TGs, triglycerides; TC, total cholesterol; LDL-C, low-density lipoprotein cholesterol; HDL-C, high-density lipoprotein cholesterol; FBG, fasting blood glucose; 2hPPG, 2-h postprandial plasma glucose; FINS, fasting insulin; HOMA-IR, homeostasis model assessment of insulin resistance; HOMA-β, homeostasis model assessment of β-cell function; EISI, early insulin secretion index; WBISI, whole body insulin sensitivity index; AUC, area under the curve; GLP-1, plasma glucagon-like peptide-1*.

* *P < 0.05*.

**Table 3:** Independent effects of dietary intakes on clinical outcomes in linear mixed-effect models.

**Outcomes**	**Total energy**		**Proportion of energy from carbohydrate**		**Proportion of energy from protein**		**Proportion of energy from fat**	
	**β (95%CI)**	***P*** **value**	**β (95%CI)**	***P*** **value**	**β (95%CI)**	***P*** **value**	**β (95%CI)**	***P*** **value**
BMI, kg/m^2^	0.00006 (−0.00004–0.0002)	0.227	0.607 (0.167–1.047)	0.007^*^	−0.177 (−1.389–1.034)	0.774	−0.808 (−1.314 −0.303))	0.002^*^
SBP, mmHg	−0.0002 (−0.001–0.0008)	0.754	1.023 (−3.312–5.357)	0.644	4.971 (−7.054–16.995)	0.418	−1.116 (−6.155–3.923)	0.664
DBP, mmHg	0.0002 (−0.0005–0.0009)	0.654	1.580 (−1.545–4.704)	0.322	−2.051 (−10.765–6.662)	0.644	−2.884 (−6.531–0.762)	0.121
TC, mmol/L	0.00002 (−0.00006–0.0001)	0.588	0.196 (−0.183–0.576)	0.311	0.348 (−0.703–1.400)	0.516	−0.244 (−0.683–0.194)	0.274
TG, mmol/L	0.00005 (−0.0001–0.0002)	0.588	−0.442 (−1.296–0.412)	0.31	0.041 (−2.351–2.432)	0.973	0.289 (−0.698–1.275)	0.566
HDL-C, mmol/L	−0.00001 (−0.00004–0.000009)	0.23	−0.085 (−0.191–0.021)	0.115	0.005 (−0.287–0.298)	0.972	0.101 (−0.021–0.224)	0.106
LDL-C, mmol/L	−0.00002 (−0.00009–0.00005)	0.568	0.209 (−0.108–0.526)	0.196	0.345 (−0.532–1.223)	0.44	−0.017 (−0.384–0.351)	0.93
FBG, mmol/L	0.0002 (0.00008–0.0003)	0.001^*^	0.322 (−0.192–0.836)	0.219	0.794 (−0.644–2.233)	0.279	−0.770 (−1.370 −0.170)	0.012^*^
HbA1c, %	0.00006 (−0.00004–0.0002)	0.239	0.685 (0.251–1.120)	0.002^*^	0.187 (−0.129–1.404)	0.763	−0.969 (−1.476–−0.462)	<0.001^*^
2hPPG, mmol/L	0.0004 (0.0002–0.0007)	0.001^*^	1.255 (0.103–2.407)	0.033^*^	−0.576 (−3.787–2.636)	0.725	−1.954 (−3.291 −0.617)	0.004^*^
FINS, μIU/Ml	0.0003 (−0.0006–0.001)	0.553	1.990 (−2.047–6.028)	0.334	3.253 (−9.051–13.756)	0.686	−2.031 (−6.739–2.677)	0.398
EISI	0.0004 (−0.0009–0.002)	0.551	−1.726 (−7.998–4.546)	0.59	10.430 (−7.798–28.657)	0.262	1.042 (−6.212–8.296)	0.778
AUC for plasma GLP-1, pmol × min	−0.007 (−0.189–0.175)	0.942	86.2 (−720.4–892.8)	0.834	−1,029.6 (−3,311.8–1,252.6)	0.377	249.8 (−702.2–1,201.9)	0.607
AUC for glucagon, pg/mL × min	−0.226 (−0.733–0.282)	0.384	−688.5 (−2,963.9–1,586.8)	0.553	−2,632.0 (−8,957.6–3,693.7)	0.415	2,371.9 (−272.3–5,016.2)	0.079
AUC for serum insulin, μIU/mL × min	0.083 (−0.118–0.285)	0.418	1,090.1 (176.6–2,003.6)	0.019^*^	200.1 (−2,370.5–2,770.8)	0.879	−1,135.1 (−2,193.8 −76.4))	0.036^*^
HOMA-IR	0.0001 (−0.0002–0.0005)	0.399	0.907 (−0.550–2.363)	0.222	0.960 (−3.149–5.069)	0.647	−0.887 (−2.598–0.815)	0.307
HOMA-β	0.0008 (−0.008–0.010)	0.864	15.773 (−23.861–55.408)	0.435	4.694 (−108.3–117.7)	0.935	−10.021 (−56.205–36.164)	0.671
WBISI	−0.0002 (−0.0006–0.0002)	0.227	−1.736 (−3.538–0.061)	0.058	1.819 (−3.230–6.869)	0.48	2.673 (0.059–4.758)	0.012^*^

*Models adjusted for age, sex, time of intervention, and duration of diabetes*.

* *P < 0.05*.

*BMI, body mass index; SBP, systolic blood pressure; DBP, diastolic blood pressure; TGs, triglycerides; TC, total cholesterol; LDL-C, low- density lipoprotein cholesterol; HDL-C, high-density lipoprotein cholesterol; FBG, fasting blood glucose; 2hPPG, 2-h postprandial plasma glucose; FINS, fasting insulin; HOMA-IR, homeostasis model assessment of insulin resistance; HOMA-β, homeostasis model assessment of β-cell function; EISI, early insulin secretion index; WBISI, whole body insulin sensitivity index; AUC, area under the curve; GLP-1, plasma glucagon-like peptide-1*.

**Figure 4:**
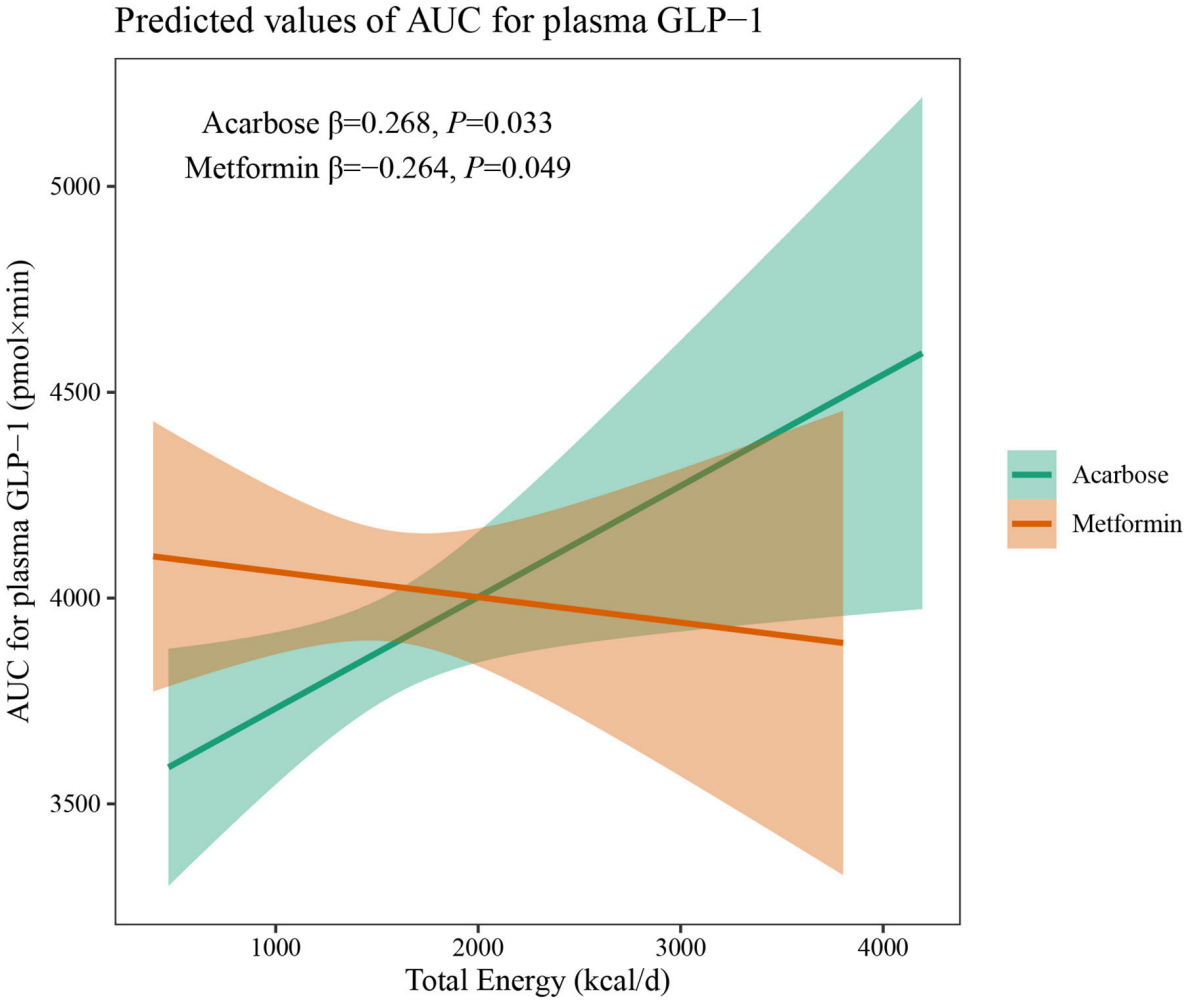
Differential associations between total energy intake and AUC for plasma GLP-1 in metformin and acarbose groups. The β value adjusted for age, sex, time of intervention, and duration of diabetes.

**Figure 5:**
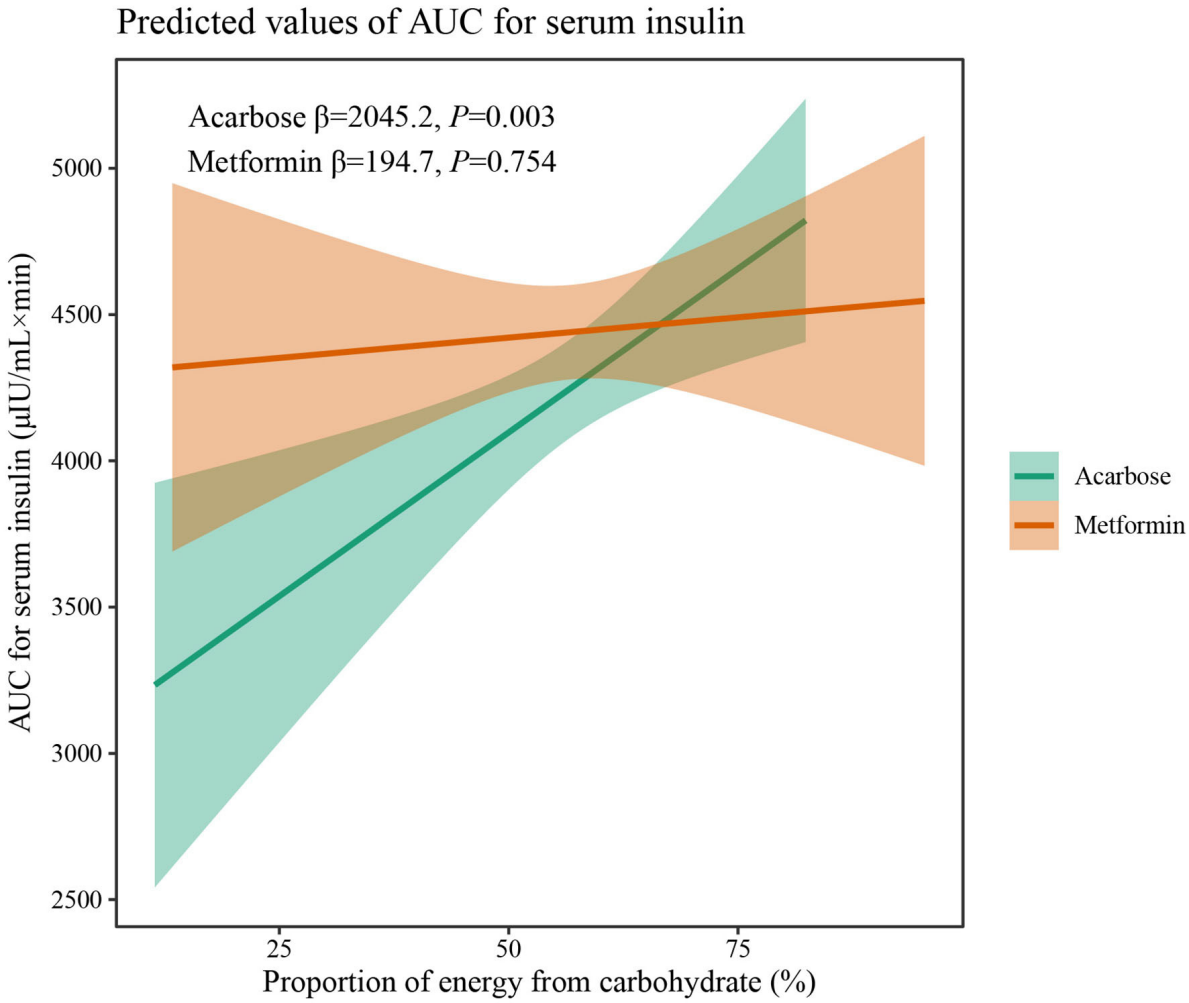
Differential associations between proportion of energy from carbohydrate intake and AUC for serum insulin in metformin and acarbose groups. The β value adjusted for age, sex, time of intervention, and duration of diabetes.

**Figure 6:**
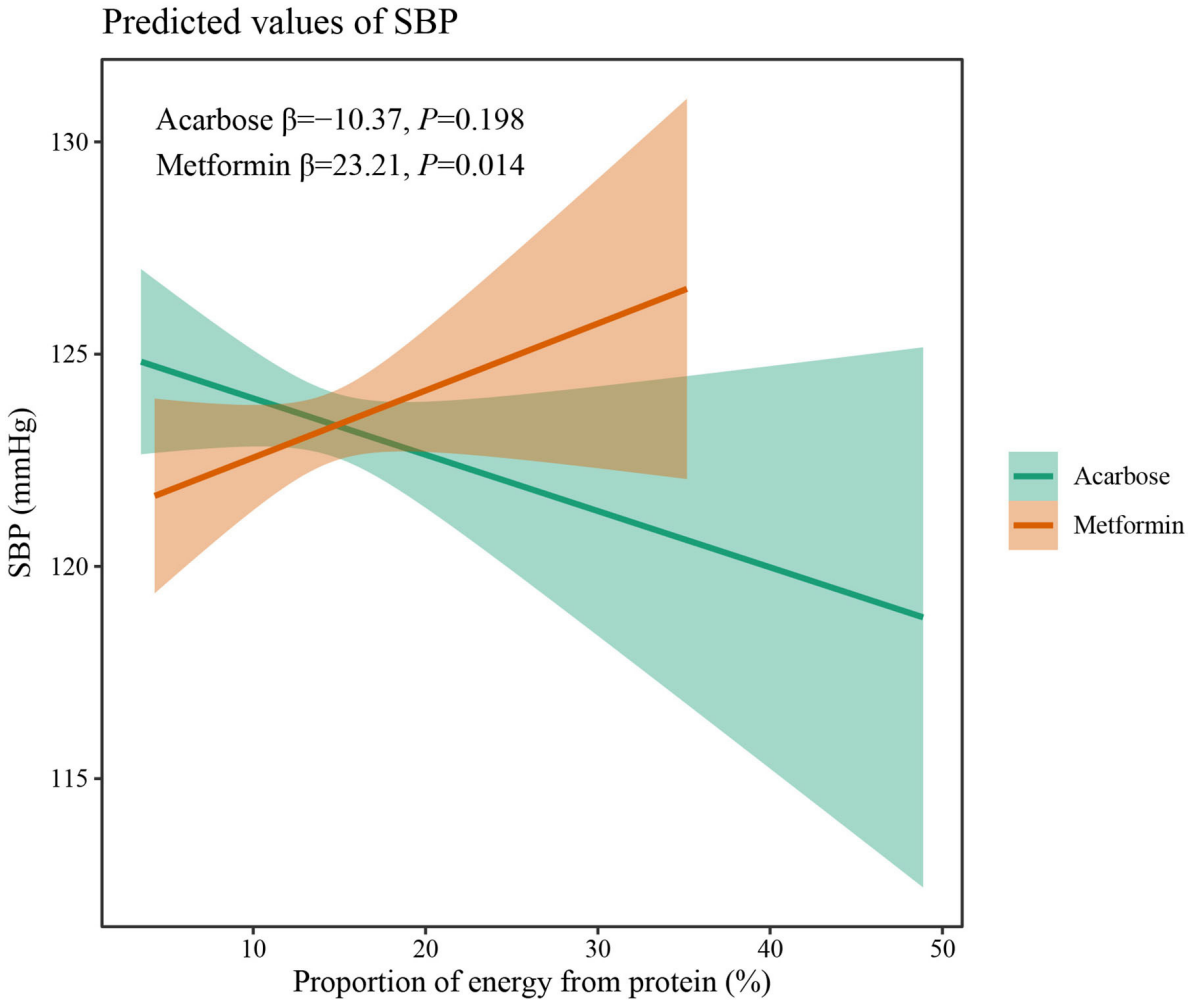
Differential associations between proportion of energy from protein intake and SBP in metformin and acarbose groups. The β value adjusted for age, sex, time of intervention, and duration of diabetes.

The results of two-way interaction analysis of the interactive effect of two drug classes with dietary intakes of total energy and macronutrients on clinical outcomes are shown in Table 4. The following was found: the interaction terms of total energy × drug therapy were associated with lower AUC for plasma GLP-1 (β = −0.335, 95% CI: −0.665 to −0.004, *P* = 0.047); the proportion of energy from carbohydrate × drug therapy was associated with lower AUC for serum insulin (β = −1,885.8, 95% CI: −3,709.7 to −62.0, *P* = 0.043); the proportion of energy from protein × drug therapy was associated with higher SBP (β = 31.71, 95% CI: 7.77–55.65, *P* = 0.009). Given the interactive effect of drug therapy with dietary intakes of total energy and macronutrients, subgroup analyses were performed on two drug classes to investigate the associations between dietary intakes and clinical outcomes (Figures 4–6). Figure 4 shows that a higher total energy intake was significantly associated with higher AUC for plasma GLP-1 (β = 0.268, *P* = 0.033) among acarbose users but with lower AUC for plasma GLP-1 among metformin users (β = −0.264 acarbose, *P* = 0.049). Additionally, it was observed that there was a significant association between higher intake of carbohydrates and AUC for serum insulin (β = 2,045.2, *P* = 0.003) in the acarbose group, but the association was not statistically significant in the metformin group (β = 194.7, *P* = 0.754, Figure 5). However, among acarbose users, no significant association was detected between dietary intake of protein and SBP, but significantly positive association was detected among metformin users (β = 23.21, *P* = 0.014, Figure 6).

**Table 4:** Diet-drug interaction on clinical outcomes in linear mixed-effect models.

**Outcomes**	**Total energy × Drug therapy**		**Proportion of energy from carbohydrate × Drug therapy**		**Proportion of energy from protein × Drug therapy**		**Proportion of energy from fat × Drug therapy**	
	**β (95%CI)**	***P*** **value**	**β (95%CI)**	***P*** **value**	**β (95%CI)**	***P*** **value**	**β (95%CI)**	***P*** **value**
BMI, kg/m^2^	0.00002 (−0.0002–0.0002)	0.858	0.149 (−0.729–1.027)	0.739	−1.012 (−3.644–1.621)	0.451	−0.095 (−1.177–0.987)	0.864
SBP, mmHg	0.0009 (−0.0010–0.0027)	0.348	−6.269 (−14.741–2.202)	0.147	31.71 (7.77–55.65)	0.009^*^	4.660 (−5.087–14.407)	0.349
DBP, mmHg	0.00016 (−0.0012–0.0015)	0.815	−1.288 (−7.346–7.770)	0.677	5.257 (−12.107–22.620)	0.533	1.758 (−5.256–8.773)	0.623
TC, mmol/L	0.00002 (−0.0001–0.0002)	0.789	0.420 (−0.328–1.168)	0.271	0.468 (−1.703–2.639)	0.673	0.018 (−0.866–0.901)	0.969
TG, mmol/L	0.00005 (−0.0003–0.0004)	0.810	1.165 (−0.541–2.871)	0.181	4.048 (−0.764–8.861)	0.099	−0.628 (−2.586–1.330)	0.529
HDL-C, mmol/L	−0.0000004 (−0.00005–0.00005)	0.985	−0.015 (−0.222–0.193)	0.888	−0.041 (−0.627–0.545)	0.890	−0.019 (−0.257–0.219)	0.875
LDL-C, mmol/L	−0.00004 (−0.0002–0.00009)	0.541	−0.105 (−0.729–0.519)	0.741	−0.888 (−2.652–0.876)	0.324	0.205 (−0.513–0.922)	0.576
FBG, mmol/L	0.0001 (−0.0001–0.0003)	0.364	−0.086 (−1.250–1.077)	0.885	−1.242 (−4.607–2.124)	0.470	−0.129 (−1.476–1.218)	0.852
HbA1c, %	−0.000001 (−0.0002–0.0002)	0.991	0.061 (−0.886–1.008)	0.900	−0.080 (−2.813–2.653)	0.954	−0.232 (−1.329–0.865)	0.679
2hPPG, mmol/L	−0.00007 (−0.0006–0.0004)	0.772	−0.905 (−3.450–1.640)	0.486	−2.333 (−9.578–4.913)	0.528	1.284 (−1.654–4.221)	0.392
FINS, μIU/Ml	0.0003 (−0.0013–0.0020)	0.747	−6.760 (−14.675–1.154)	0.094	4.027 (−18.854–26.908)	0.730	2.957 (−6.143–12.057)	0.524
EISI	0.0015 (−0.0011–0.0042)	0.253	4.768 (−7.760–17.297)	0.456	−10.995 (−47.379–25.388)	0.554	−2.613 (−17.023–11.797)	0.722
AUC for plasma GLP-1, pmol × min	−0.335 (−0.665 −0.004)	0.047^*^	−6.446 (−1,588.6–1,576.7)	0.994	−122.01 (−4,723.1–4,479.1)	0.959	143.5 (−1,690.9–1,977.9)	0.878
AUC for glucagon, pg/mL × min	0.186 (−0.772–1.144)	0.704	938.8 (−3,550.4–5,428.0)	0.682	−6,141.4 (−18,886.8–6,604.0)	0.345	−1,162.8 (−6,322.2–3,996.6)	0.659
AUC for serum insulin, μIU/mL × min	−0.345 (−0.732–0.042)	0.081	−1,885.8 (−3,709.7 −62.0)	0.043^*^	−2,000.8 (−7,225.9–3,224.4)	0.453	1,670.1 (−427.7–3,767.9)	0.119
HOMA-IR	0.0001 (−0.0005–0.0007)	0.723	−2.056 (−4.954–0.842)	0.164	1.090 (−7.301–9.482)	0.799	0.500 (−2.837–3.838)	0.769
HOMA-β	0.0039 (−0.0125–0.0203)	0.639	−70.508 (−147.4–6.368)	0.072	67.24 (−155.97–290.45)	0.555	50.00 (−38.53–138.54)	0.268
WBISI	0.00008 (−0.0007–0.0008)	0.839	2.297 (−1.493–6.087)	0.235	−2.756 (−13.635–8.123)	0.62	−1.317 (−5.667–3.033)	0.553

*Models adjusted for age, sex, time of intervention, and duration of diabetes*.

*BMI, body mass index; SBP, systolic blood pressure; DBP, diastolic blood pressure; TGs, triglycerides; TC, total cholesterol; LDL-C, low-density lipoprotein cholesterol; HDL-C, high-density lipoprotein cholesterol; FBG, fasting blood glucose; 2hPPG, 2-h postprandial plasma glucose; FINS, fasting insulin; HOMA-IR, homeostasis model assessment of insulin resistance; HOMA-β, homeostasis model assessment of β-cell function; EISI, early insulin secretion index; WBISI, whole body insulin sensitivity index; AUC, area under the curve; GLP-1, plasma glucagon-like peptide-1*.

* *P < 0.05*.

## Discussion

Recent studies have drawn attention to the potential interactive effects of chronic medication use with nutrient intakes on chronic noncommunicable diseases (20, 21). Drug-nutrient interactions are of great clinical and public health significance, especially in patients with T2DM. The treatment for T2DM involved dietary manipulation that can reduce both glucose and insulin aberrant levels, and cardiovascular complications. This study first showed two classic antidiabetic medications of acarbose and metformin and their interaction with macronutrient intakes in the context of MARCH clinical trial. Among the included Chinese patients being treated for T2DM, metformin and acarbose mainly exerted different interactive effects with dietary energy, carbohydrate, and protein intakes on GLP-1 secretion, insulin release, and SBP.

The results of this study have shown that the daily intake of total energy, carbohydrates, protein, and fat for included newly diagnosed patients with T2DM fell into the normal range before treatment, and total energy intake remained relatively stable in both two groups during treatment. Even so, independent and positive associations were still found between total energy and FBG, as well as 2hPPG during treatment, suggesting additional benefits of glycemic control from total energy restriction even under antidiabetic oral agent therapy, which was also observed among Korean patients (22). Meanwhile, different oral drugs may change the association between total energy intake and treatment outcomes due to different mechanisms of action related to the gastrointestinal tract. In the subgroup analysis, total energy intake and AUC for plasma GLP-1 showed inverse associations under metformin and acarbose treatment, indicating that total energy intake stimulates distinct patterns of GLP-1 release. The incretin hormone of GLP-1 plays a critical role in regulating glucose homeostasis and dietary intakes of nutrients are a primary stimulus to the release of intestinal GLP-1 (23). In generally, carbohydrate, lipid, and protein digestion, also known as sources of total energy, can induce postprandial glycemic and GLP-1 secretion through different pathways, to promote the absorption of glucose and maintain the level of blood glucose (24). Consequently, strategies to slow down the digestion of these macronutrients are needed to achieve a sustained release of GLP-1 for improvement of the glucose homeostasis. It has been confirmed that postprandial GLP-1 secretion under acarbose treatment is stimulated in patients with T2DM by induction of transfer of carbohydrates to the distal parts of the intestine (25). Therefore, under acarbose treatment, a sustained release of GLP-1 can still be achieved even following an increase in total energy intake by slowing down the digestion and absorption of dietary carbohydrates in small intestine, suggesting a positive association between total energy intake and AUC for plasma GLP-1. However, under metformin treatment, a higher total energy intake was associated with lower AUC for plasma GLP-1. It has also been showed that metformin increases plasma GLP-1 concentrations in healthy controls and patients with T2DM (26). Nevertheless, it has been reported that metformin does not directly stimulate GLP-1 secretion *in vitro*, suggesting that its effect observed *in vivo* are indirect (27). It may be speculated that the GLP-1 secretion induced by metformin can be disturbed by total energy intake, which deserves further investigation.

Contrary to relatively stable intake of total energy, carbohydrate intake significantly decreased at 24 weeks but increased at 48 weeks in the acarbose group and significantly increased at 48 weeks in the metformin group. Considering that the staple food is rich in carbohydrate in most Asian countries, the changes in quantity of dietary carbohydrates indicated a gradually loose restriction on carbohydrate and a relative imbalance between dietary desires and adherence to diet modification in disease management. Dietary carbohydrates are one of fundamental macronutrients in terms of their capacities to affect blood glucose and insulin levels (28). In linear mixed-effect models, higher intake of carbohydrates was independently associated with higher BMI, HbA1c, 2hPPG, and AUC for serum insulin, which was also observed in Saudi patients (29), suggesting that carbohydrate restriction is essential for the achievement of good glycemic control, weight loss, and reduced insulin secretion. In addition, under acarbose and metformin treatment, significant and comparable effects on reduction of BMI and HbA1c were still achieved, while under acarbose treatment, significantly lower postprandial blood glucose was achieved after 48 weeks, and AUC for serum insulin decreased to a greater extent than under metformin treatment, demonstrating that acarbose is superior to metformin in reducing postprandial hyperglycemia and hyperinsulinemia in patients with T2DM consuming an Asian diet. Meanwhile, the association between dietary carbohydrates and AUC for serum insulin was only significant under acarbose but not metformin treatment, which may contradict the insulin-sparing effect of acarbose (30). However, it also reflected, from another perspective, beneficial effects of acarbose on preserving β-cell function (31).

Finally, synergetic effects of protein intake and metformin treatment on lowering SBP were found. Some intervention studies have proved that a high protein intake can significantly decrease SBP and DBP in patients with T2DM (32). Furthermore, Garnett et al. (33) also observed that the SBP and DBP of metformin-treated prediabetic adolescents significantly decreased over time when receiving an increased-protein diet, all of which may suggest that consuming more proteins may help metformin users feel more at ease over time.

Since total energy and protein intake remained relatively stable in this study, the increase in the proportion of energy from carbohydrates was accompanied by the decrease in energy from fat. Not surprisingly, this led to the positive associations of higher dietary fat intake with better glycemic control, weight loss, the improvement of insulin secretion, and higher WBISI, an indicator of insulin sensitivity. In spite of contradicting the results reported in most studies that a reduction in dietary fat can promote weight loss benefiting blood lipid and glucose profiles (34–36), the result of association between a relatively higher fat intake and better treatment outcomes may reflect interactions between macronutrients, which make it difficult to interpret the individual contributions of each source to clinical outcomes. Moreover, aside from the quantity of dietary fat and carbohydrates, growing evidence suggests that the quality of dietary fat and carbohydrates (with a preference for foods with natural unsaturated fat, high fiber, low glycemic index, and whole grain instead of those with trans or saturated fat) are stronger determinants of the effects of diet on metabolic control than the quantity of each macronutrient in the diet (37), which may be further investigated in the following studies.

The MARCH trial provided evidence that both acarbose and metformin have a similar effect as initial therapy on HbA1c reduction in Chinese patients with T2DM. Shortly after, a meta-analysis systematically searched the Chinese and English literature for eligible randomized controlled trials to compare glucose-lowering effects of metformin and acarbose, which also implied that the effect of metformin is at least as good as that of acarbose (38). In this study, it was also found that acarbose treatment was not inferior to metformin treatment in view of most clinical outcomes. However, linear mixed-effect models showed metformin treatment was associated with higher TG and 2hPPG but lower LDL-C compared with acarbose treatment. Although none of these outcomes was affected by interaction of drug and dietary macronutrients, the superior effect of acarbose on postprandial plasma glucose still demonstrated a stronger response to dietary intakes compared with metformin. The research results only showed three significant interactions. Perhaps, due to a relatively shorter observation, there were limited changes in dietary macronutrients over time and relatively overwhelming effects of intensive drug therapy. Although there is little evidence of drug–diet interaction, the potential mechanism may be explainable or incidental in this study. Therefore, great importance should be attached to drug–diet interaction, and further investigation should be conducted in physiological studies and clinical solutions.

To the best of our knowledge, this study is the first of its kind to document the interaction of between pharmacotherapies and dietary intakes of macronutrients in patients with T2DM. However, there are several limitations to consider. First, all participants were Chinese patients newly diagnosed with T2DM, which may limit the application to other patients to some extent, and the association may be largely different in patients with longer duration of diabetes or following Western dietary patterns. However, the results may be applicable to other Chinese populations with diabetes, with characteristics similar to the sample in this study. Second, only limited food and beverage items were evaluated by the 24-h dietary recall method, which may lead to an underestimation of energy and macronutrients intake. Third, measurement errors in self-reported dietary behaviors were inevitable. Finally, the fat type and quality of carbohydrates were not considered in this study.

## Conclusion

The MARCH trail provided a unique opportunity to investigate the drug–diet interactive effect on treatment outcomes in patients with T2DM. Metformin and acarbose mainly exerted different interactive effects with dietary macronutrients on GLP-1 secretion, insulin release, and SBP, suggesting that the complexities of drug-diet therapies may confer distinct benefits of glycemic control.

## Data Availability Statement

The raw data supporting the conclusions of this article will be made available by the authors, without undue reservation.

## Ethics Statement

The studies involving human participants were reviewed and the protocol was approved by an Ethics Committee from each clinical site. The patients/participants provided their written informed consent to participate in this study.

## Author Contributions

GW and JL conceptualized and designed the study, obtained, and supervised data collection. YA and YL conducted the data analysis and drafted the manuscript. NB, XD, and XC helped perform data processing and analysis. All authors contributed to the article and approved the submitted version.

## Conflict of Interest

The authors declare that the research was conducted in the absence of any commercial or financial relationships that could be construed as a potential conflict of interest.

## Publisher's Note

All claims expressed in this article are solely those of the authors and do not necessarily represent those of their affiliated organizations, or those of the publisher, the editors and the reviewers. Any product that may be evaluated in this article, or claim that may be made by its manufacturer, is not guaranteed or endorsed by the publisher.
